# Clinical implications of DLL4 expression in gastric cancer

**DOI:** 10.1186/1756-9966-32-46

**Published:** 2013-07-30

**Authors:** Sumiya Ishigami, Takaaki Arigami, Yoshikazu Uenosono, Hiroshi Okumura, Hiroshi Kurahara, Yasuto Uchikado, Tetsuro Setoyama, Yoshiaki Kita, Yuko Kijima, Yuka Nishizono, Akihiro Nakajo, Tetsuro Owaki, Shinichi Ueno, Shoji Natsugoe

**Affiliations:** 1 Department of Digestive Surgery, Breast and Thyroid Surgery, Kagoshima University School of Medicine, Sakuragaoka, Kagoshima 890-8520, Japan

**Keywords:** Delta-like ligand 4, Lymphatic invasion, Lymph node metastases, Gastric cancer, Peritumoral stroma

## Abstract

**Background:**

Delta-like ligand 4 (DLL4)-Notch signaling plays a key role in tumor neovascular development and angiogenesis during tumor growth. The clinical significance of DLL4 expression in gastric cancer has not been clarified.

**Methods:**

Gastric cancer cell lines and 180 gastric cancer patients were enrolled. DLL4 expression in gastric cancer cells and stroma was identified and evaluated immunohistochemically. The association between DLL4 and clinicopathological factors was also assessed.

**Results:**

DLL4 expression was identified in the cellular membrane and cytoplasm of gastric cancer cells by immunoblotting and immunohistochemical staining. DLL4 positivity in cancer cells and stroma was found in 88 (48%) and 41 (22%) of the 180 gastric cancer patients respectively. Both cancer and stromal DLL4 expression significantly correlated with more advanced tumor depth, nodal involvement, and lymphatic and venous invasion. A strongly positive association between cancerous and stromal DLL4 expression was identified (p < 0.01). Both cancerous and stromal DLL4 expression were prognostic markers in gastric cancer as determined by univariate analysis.

**Conclusions:**

Cancerous and stromal DLL4 expression was found in 48% and 22% in gastric cancer, and significantly affected postoperative clinical outcomes. Cancerous and stromal DLL4 expression may be an effective target of anti-DLL4 treatment in gastric cancer.

## Introduction

Gastric cancer is one of the major causes of cancer-related deaths worldwide, especially in East Asia [1-3]. When gastric cancer is diagnosed and treated in the early stages, the prognosis is good. However, some patients have an unfavorable postoperative outcome, despite receiving curative surgery. In addition, gastric cancer patients with distant metastases cannot undergo curative surgery. The recent development of novel anticancer agents in unresectable gastrointestinal cancer has improved clinical outcomes. Antiangiogenetic agents are promising for treating advanced, refractory tumors. As angiogenesis directly affects tumor growth and metastasis, it may be an important target for control of tumor progression [4,5]. Antiangiogenic agents such as bevacizumab, which target the vascular endothelial growth factor (VEGF) pathway and inhibit angiogenesis, are promising for the treatment of multiple cancers, including advanced and recurrent gastric cancer. In clinical trials, these anti-VEGF agents have been shown to prevent tumor progression and improve overall survival in colorectal, breast, and lung cancer [6-8], as well as advanced gastric cancer [9,10]. Currently, a promising antiangiogenetic therapy that is unrelated to VEGF-VEGF receptor (VEGFR) signaling has been demonstrated for bevacizumab-refractory cancer.

The Notch receptors (Notch-1, -2, -3, -4) and their ligands (Delta-like ligands (DLL)-1, -2, -3, -4, and Jagged-1 and Jagged-2) are critically involved in tumor neovascularity. In particular, it has been elucidated that the Notch Delta-like ligand 4 (DLL4) regulates tumor angiogenesis [11,12], and plays key roles in tumor neovascularity [12,13]. Troise et al. reported that blocking DLL4 –Notch signaling caused nonproductive angiogenesis of tumor vessels, and drastic shrinkage of tumors in mouse models [14,15]. Moreover, a soluble form of DLL4 blocked tumor growth in both bevacizumab-sensitive and bevacizumab-resistant tumors by disrupting vascular function. Recent studies have demonstrated that DLL4 expression can be found not only in peritumoral tissues, but also in the tumor cell itself [16,17]. However, there is little published data examining DLL4 expression in gastric cancer. We used immunohistochemistry to evaluate DLL4 expression of cancer cells and stroma in gastric cancer, speculating upon the clinical impact of this expression profile.

## Materials and methods

180 gastric cancer patients (128 men, mean age 65 – range 41–85) who underwent gastrectomy at Kagoshima University Hospital between 2001 and 2004 were enrolled. None of the patients received preoperative chemotherapy. All patients underwent R0 resection with greater than D1 lymph node dissection. Clinical factors were assessed by the Japanese Classification of Gastric Carcinoma [18]. Sample collection and processing are described in greater detail in the online supplemental data. Written informed consent was obtained from all patients and the study was approved by our institutional ethics committee. This investigation conformed to the principles outlined in the Declaration of Helsinki.

### Immunohistochemistry

**Figure 1:**
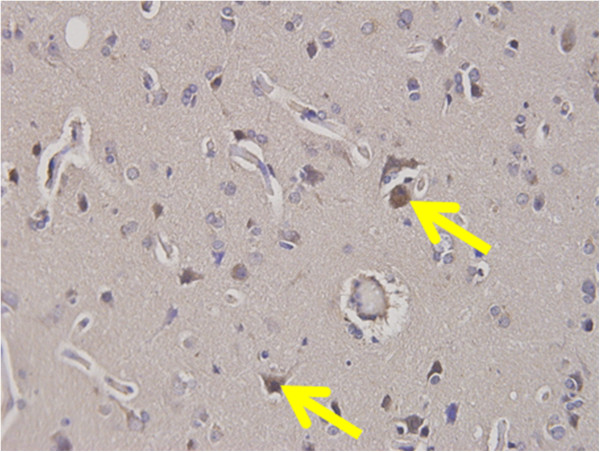
**DLL4 expression in brain tissue.** DLL4 expression was identified in the brain tissue as a positive control of DLL4.

Paraffin-embedded sections (4 μm), including tumor nests were obtained. Sections were deparaffinized and soaked in PBS prior to immunohistochemical analysis. Sections were also soaked in 3% H_2_O_2_ for 30 minutes in order to block endogenous tissue peroxidase, followed by treatment with bovine serum for 30 minutes in order to reduce nonspecific binding. The DLL4 antibody (rabbit polyclonal; ab103469; Abcam) was diluted to 1:200, and incubated at room temperature for 12 hours. Sections were rinsed in PBS and visualized by standard techniques for labeled avidin-biotin immunoperoxidase staining. Then, DLL4 was visualized using a DAB Substrate Kit. The slides were briefly counterstained with hematoxylin and mounted aqueously. Human brain tissue was used as a positive control for DLL4 expression (Figure 1). DLL4 positivity of four gastric cancer cell lines was examined by the same procedures of paraffin-embedded tissue without deparaffinization.

### Detection of DLL4 expression in gastric cancer cell lines by western blot analysis

Gastric carcinoma cell lines, MKN28, MNK45, KATO-III, and NUGC4 were purchased from the Japanese Physical and Chemical Institute, Tokyo, Japan. They were maintained in RPMI 1640 supplemented with 10% fetal bovine serum (FBS), 100 units/ml penicillin, and 100 μg/ml streptomycin at 37°C in a cell incubator. All cells were harvested by centrifugation, rinsed with phosphate buffered saline (PBS), and subjected to total protein extraction in an immunoprecipitation assay buffer lysis buffer. The separation of nuclear extract and cytoplasmic fraction was performed by a Nuclear/Cytosol Fraction Kit (K266-25 BioVison, USA). Cytoplasmic and nucleus DLL4 expression were extracted to facilitate Western blot.

Western blot analysis of DLL4 of gastric cancer cell lines was performed. Denatured proteins extracted from the nucleus and cytoplasm were separated on an SDS-polyacrylamide gel and transferred to Hybond membrane, which was then blocked overnight in 5% skimmed milk in TBS. For immunoblotting, the membrane was incubated for 15 minutes with mouse antibody against DLL4 (1:2000). Then, it was rinsed by TBST and incubated with anti-house IgG conjugated to horseradish peroxidase for 15 minutes. Bands were visualized with X-ray film (Fuji, Japan) by ECL-Plus detection reagents. After that, the membrane was washed with WB Stripping Solution (Nakarai, Tokyo, Japan) for 15 minutes and treated as described above except anti-β-actin antibody (sc-47778, Santa Cruz, 1:1000) as the internal control.

### Evaluation of DLL4 expression of cancer cells and stroma in gastric cancer

**Figure 2:**
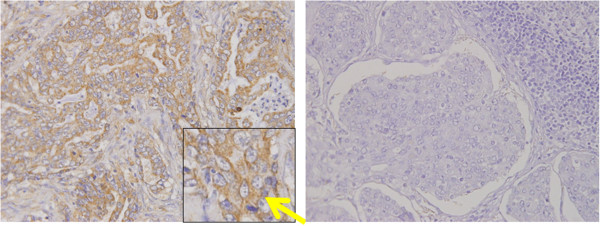
**DLL4 expression in gastric cancer cells.** Right: DLL4 expression was identified in the cellular membrane of gastric cancer cells. DLL positivity was found in the cytoplasm and cellular membrane of gastric cancer (yellow arrow). Left: DLL4 expression was not found in gastric cancer (negative control).

**Figure 3:**
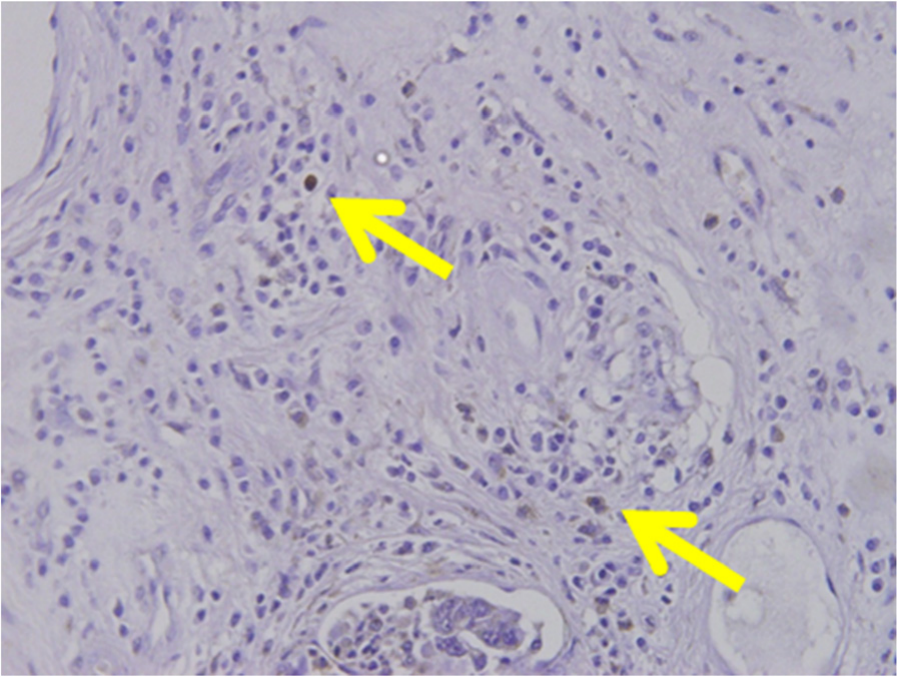
**DLL4 expression in brain and stromal cells of gastric cancer.** DLL4 positive infiltrative cells were identified in cancer stroma (yellow arrow).

DLL4 expression in cancer cell membranes was evaluated, and averaged in ten different high power magnified fields (× 400). DLL4 expression was identified in the cytoplasm and cellular membrane of cancer cells (Figure 2), and in the stromal cells (Figure 3). Ten representative tissue sections were observed by light microscropy and the percentage of DLL4 positive cancer cells was scored, averaged, and scored semiquantitatively. All immunostained slides were evaluated by two independent observers (SI and AT), who were unaware of the clinical data and disease outcome. If more than 10% of dominant staining intensity in tumor cells or stromal cells was identified, the patients were regarded as DLL4 positive. After evaluation, patients were divided into two groups according to DLL4 expression positivity. Clinicopathological factors of gastric cancer were assessed according to the General Rules of Gastric Cancer in Japan [18].

### Statistical analysis

Statistical analysis of clinical features was performed using the χ^2^-test. Survival curves were constructed using the Kaplan-Meier method, and survival differences were analyzed by the generalized Wilcoxon test. Multivariate analysis was performed to determine prognostic factors. A p-value of less than 0.05 was considered to be statistically significant.

## Results

### DLL4 expression in gastric cancer tissues

DLL4 positivity was identified in brain tissue as a positive control of DLL4 (Figure 1). DLL4 expression was primarily identified in the membranes and cytoplasm of cancer cells, regardless of tumor histology (Figure 2), as well as infiltrative cells in cancer stroma (Figure 3). 88 (49%) patients were classified as DLL4 positive (10% of DLL4 positive) group in cell lines; 41 (23%) were positive in the stroma.

### DLL4 expression in gastric carcinoma cell lines

**Figure 4:**
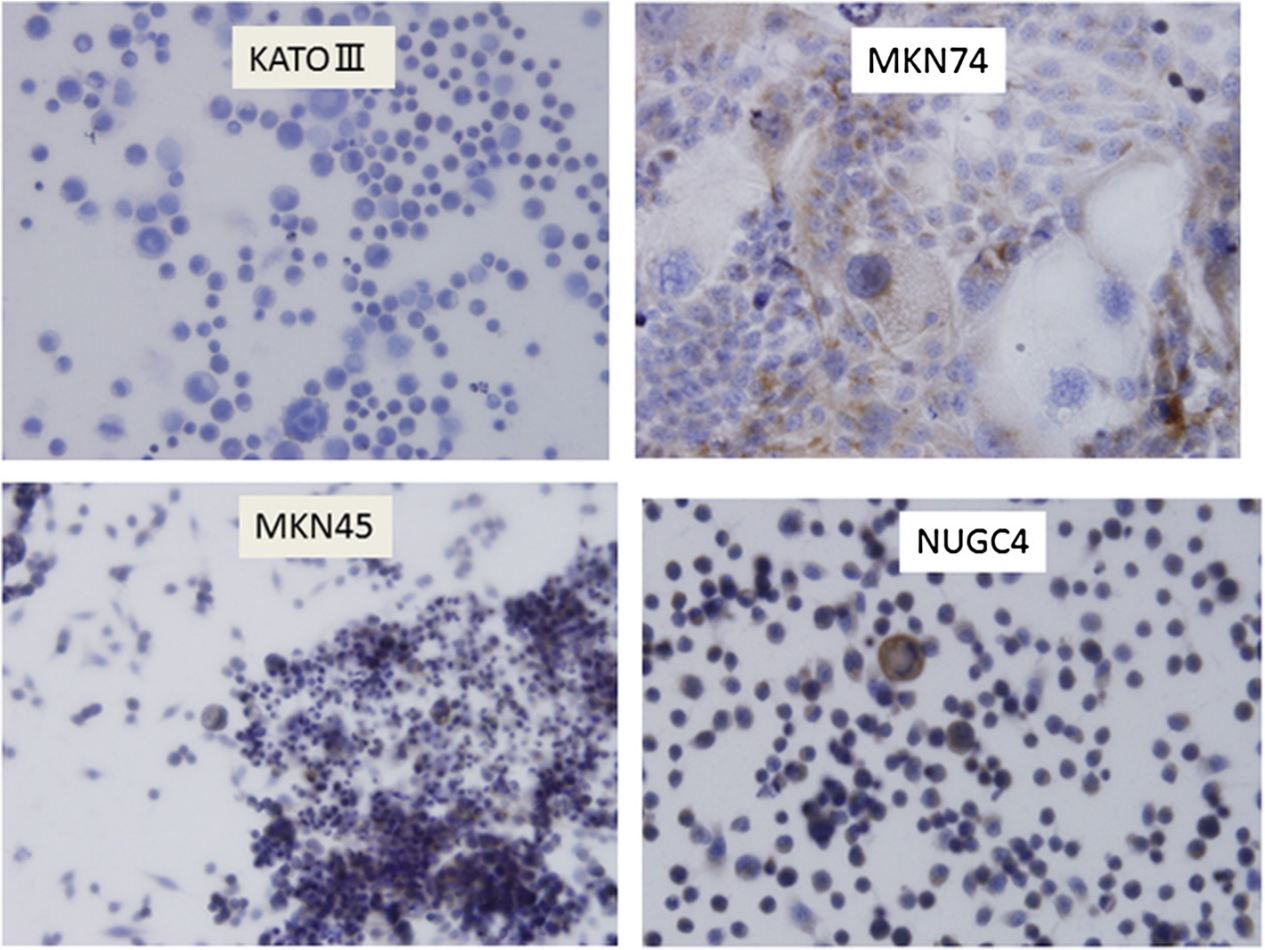
**DLL4 expression in gastric cancer cell lines.** DLL4 expression was identified in the cellular membrane and cytoplasm of gastric cancer cells.

**Figure 5:**
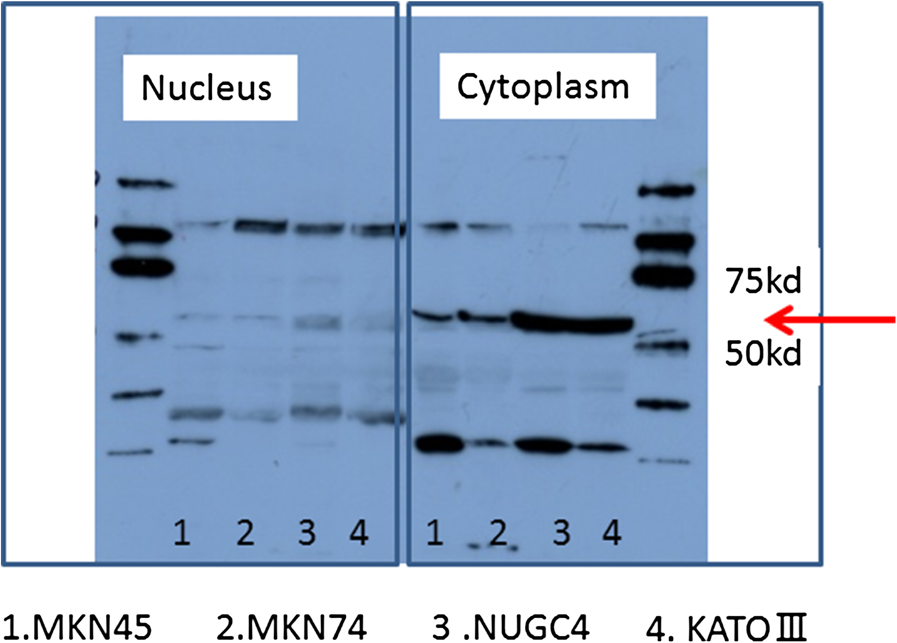
**DLL4 protein detection in gastric cancer cell lines by Western blot analysis.** DLL4 was detected in the cytoplasm of gastric cancer cell but not the nucleus in four gastric cancer cell lines.

Immunohistochemical staining showed DLL4 expression in cytoplasm of the four gastric cancer cell lines (Figure 4). Cell lysates extracted separately from the nucleus and cytoplasm in the gastric cancer cell lines were loaded and probed with anti-DLL4 antibody. DLL4 protein was identified in cytoplasm of the all gastric cancer cell lines, but not in the nucleus (Figure 5).

### Clinicopathological features of DLL4-positive group

Clinicopathologic features of DLL4-positive gastric cancers were assessed. The DLL4-positive group had a greater depth of tumor invasion (p < 0.01, p < 0.01), more lymph node metastases (p < 0.01, p < 0.05), and significantly more venous (p < 0.05, n.s.) and lymphatic invasion (p < 0.01, p < 0.01 respectively) in not only the cancer cell but also stroma (Table 1, Table 2). However, there was no significant difference in other clinical factors.

**Table 1:** Association between cancerous DLL4 expression and clinical factors in 180 gastric cancer

**Clinical**		**(n)**	**DLL4 positive**	**DLL4 negative**	**p value**
**Factors**			**(n = 88)**	**(n = 92)**	
Sex	Male	128	62	66	
	Female	52	26	26	n.s.
Age			64.2	66.1	n.s.
T factor	T1	72	11	61	
	T2	54	41	13	
	T3	44	28	16	p < 0.01
	T4	10	8	2	
N factor	N0	93	24	69	
	N+	87	64	23	p < 0.01
Lymphatic invasion	No	78	18	60	
	Yes	102	70	32	p < 0.01
Venous invasion	No	102	31	71	
	Yes	78	57	21	p < 0.05
Histology	Differentiated	98	47	51	
	Undifferentiated	82	41	41	n.s.

**Table 2:** Association between stromal DLL4 expression and clinical factors in 180 gastric cancer

**Clinical**		**(n)**	**DLL4 positive**	**DLL4 negative**	**p value**
**Factors**			**(n = 41)**	**(n = 139)**	
Sex	Male	128	28	100	
	Female	52	13	39	n.s.
Age			63.1	65.7	n.s.
T factor	T1	72	6	66	
	T2	54	14	40	
	T3	44	17	27	p < 0.01
	T4	10	4	6	
N factor	N0	93	15	79	p < 0.01
	N+	87	26	60	
Lymphatic invasion	No	78	10	68	p < 0.01
	Yes	102	31	71	
Venous invasion	No	102	14	88	
	Yes	78	37	51	n.s.
Histology	Differentiated	98	23	75	
	Undifferentiated	82	18	64	n.s.

### Prognostic impact of DLL4 positivity in gastric cancer

**Figure 6:**
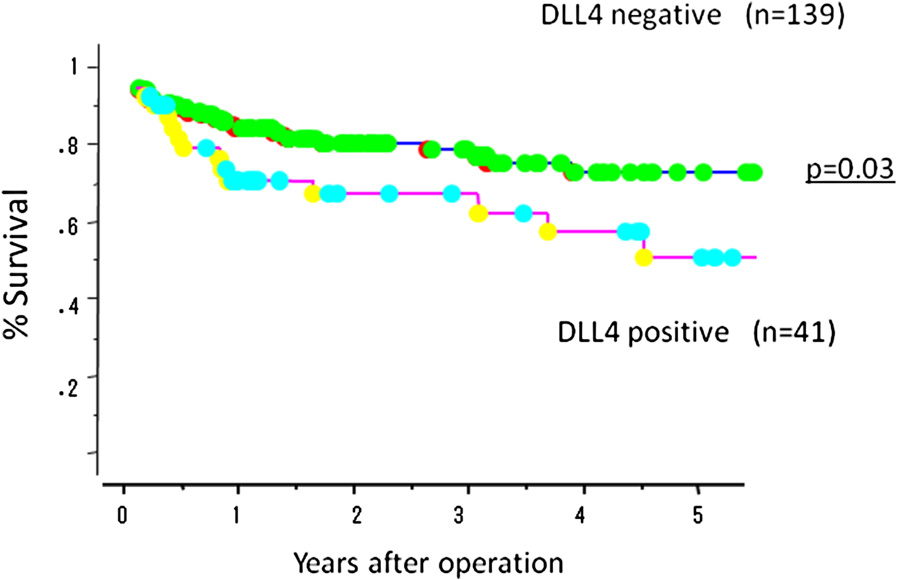
**Overall survival of 180 gastric cancer patients according to DLL4 expression in cancer cell.** DLL4-positive patients had significantly poorer survival than DLL4-negative patients (p < 0.01).

**Figure 7:**
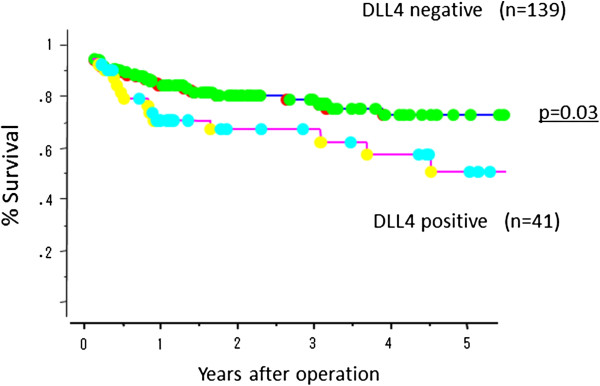
**Overall survival of 180 gastric cancer patients according to DLL4 expression in cancer stroma.** DLL4-positive patients in cancer stroma had significantly poorer survival than DLL4-negative patients (p = 0.03).

Overall surival of gastric cancer in the absence or presence of DLL4 expression were evaluated by univariate and multivariate analyses. The DLL4-positive cancer group had a significantly poorer survival than the DLL4-negative group (p < 0.01; Figure 6). Moreover, the DLL4-positive stroma group also had a significantly poorer survival than negative group (p = 0.03; Figure 7). By univariate analysis, tumor depth, nodal involvement, lymphatic invasion, and DLL4 positivity were found to be significant prognostic markers. However, multivariate analysis did not demonstrate DLL4 to be an independent prognostic marker for survival (Table 3).

**Table 3:** Univariate and multivariate analysis of survival with clinical factors including DLL4 expression

**Factors**	**Univariate**	**Multivariate**		
	**p value**	**p value hazard ratio**		**95% CI**
Cancerous DLL4	<0.01	=0.11		
Stromal DLL4	<0.05	=0.22		
Depth of invasion	<0.01	<0.01	0.35	0.16–0.72
Nodal involvement	<0.01	<0.01	0.09	0.02–0.47
Lymphatic invasion	<0.05	=0.97		
Venous invasion	<0.05	=0.		

## Discussion

Previously, expression in cancerous tissue was thought to be limited to the endothelial cells of peritumoral vessels. However, recent reports have shown a strong association of DLL4 expression in the cellular membrane of tumor cells themselves [19-21]. Therefore, to more accurately evaluate DLL4 function, its expression must be examined in both the peritumoral vasculature and cancer cells.

In the current study, cancerous and stromal DLL4 expression were found in 49% and 23% of gastric cancer patients, which lower than that of colorectal cancer [16]. Moreover, stromal DLL4 expression was not as remarkable as previously reported in breast cancer [22]; therefore, the pattern of DLL4 expression in gastric cancer may be different from that of breast cancer.

Experimentally, DLL4 expression in cancer cells has been previously analyzed. Li *et al*. showed that DLL4 was upregulated in human glioblastoma [23]; DLL4 expression in tumor cells activated Notch signaling in endothelial cells; in addition, DLL4 overexpression in glioma cells led to tumor proliferation, angiogenesis, metastasis, and resistance to hormonal and chemotherapy. The activated Notch1 signal pathway has been shown to be involved with gastric cancer progression. Yeh et al. showed that activation of Notch1 receptor promoted colony forming ability and tumor growth of cell lines in gastric cancer [24]. Thus, DLL4 expression in the tumor cells was functionally active, and appears to be consistent with our clinical data.

In our study, DLL4-positive cancer had more lymph node metastases and severe lymphatic invasion. Moreover, stromal DLL4 expression also correlated with tumor spread. We found a significant correlation between cancerous and stromal DLL4 expression; thus, DLL4 may be associated with lymphatic metastasis, consistent with what has been shown in other cancers. Jubb et al. investigated DLL4 expression in metastatic breast cancer after VEGF treatment, and found anti-VEGF agents to be efficacious in treating DLL4-positive cancers [22] – suggesting DLL4 to be a good target for antiangiogenic therapies. Moreover, Patel et al. showed that DLL4 was closely associated with vascular differentiation in bladder cancer; DLL4 appeared to be a novel target for antiangiogenic treatment in this scenario as well [25,26]. For tumors in which anti-VEGF treatment is less effective, Nogueira et al. suggested that blocking DLL4 signaling might be a promising strategy [15].

As a prognostic marker, DLL4 positivity contributed to poor clinical outcomes in gastric cancer, which was similar to reports by Jubb et al. [17]. By multivariate analysis, DLL4 was not found to be an independent prognostic marker, which may be influenced by the strong association with lymph node metastasis. Interestingly, DLL4 expression in other cancers is not always associated with a poor clinical prognosis. For example, Donnem et al. demonstrated that DLL4 positivity was a good prognostic marker in lung adenocarcinoma [27], different from our results. Organ specificity in the evaluation of DLL4 expression of the tumor tissues should be considered.

## Conclusions

In conclusions, cancerous and stromal DLL4 expression may be one of the angiogenesis-related prognostic markers in gastric cancer. Since this protein plays a key role in angiogenesis, future studies are required to determine if antiangiogenic therapy will be useful in DLL4-expressing gastric cancer. Cancerous and stromal DLL4 expression may be a good target for anti-DLL4 therapy in gastric cancer.

## Competing interests

The authors declare that they have no competing interest.

## Authors’ contribution

SI and AT wrote the manuscript. SN, YU and HO contributed conceptual information and edited the manuscript. All authors read and approved the final manuscript.
